# A novel CPU/GPU simulation environment for large-scale biologically realistic neural modeling

**DOI:** 10.3389/fninf.2013.00019

**Published:** 2013-10-02

**Authors:** Roger V. Hoang, Devyani Tanna, Laurence C. Jayet Bray, Sergiu M. Dascalu, Frederick C. Harris

**Affiliations:** ^1^Brain Computation Laboratory, Department of Computer Science and Engineering, University of Nevada, RenoNV, USA; ^2^Brain Computation Laboratory, Department of Bioengineering, George Mason University, FairfaxVA, USA

**Keywords:** neocortical simulator (NCS), CPU/GPU simulation, leaky integrate-and-fire neurons, izhikevich neurons, biologically realistic, large-scale modeling

## Abstract

Computational Neuroscience is an emerging field that provides unique opportunities to study complex brain structures through realistic neural simulations. However, as biological details are added to models, the execution time for the simulation becomes longer. Graphics Processing Units (GPUs) are now being utilized to accelerate simulations due to their ability to perform computations in parallel. As such, they have shown significant improvement in execution time compared to Central Processing Units (CPUs). Most neural simulators utilize either multiple CPUs or a single GPU for better performance, but still show limitations in execution time when biological details are not sacrificed. Therefore, we present a novel CPU/GPU simulation environment for large-scale biological networks, the NeoCortical Simulator version 6 (NCS6). NCS6 is a free, open-source, parallelizable, and scalable simulator, designed to run on clusters of multiple machines, potentially with high performance computing devices in each of them. It has built-in leaky-integrate-and-fire (LIF) and Izhikevich (IZH) neuron models, but users also have the capability to design their own plug-in interface for different neuron types as desired. NCS6 is currently able to simulate one million cells and 100 million synapses in quasi real time by distributing data across eight machines with each having two video cards.

## Introduction

Many different scales of experiments in neuroscience research attempt to clarify the complex functions of the nervous system. From the genetics of single molecules to the behavioral research of cognitive neuroscience, studies lead to a better understanding of neural networks, such as the brain. When *in vivo* and *in vitro* experiments are impossible to perform due to the complexity of structures, computational neuroscience provides new opportunities. Its unique access to any brain region as well as its different levels of abstraction allow biologically-realistic neural simulations, and thus additional neuroscience findings. However, neural simulations have always involved a trade-off between execution time and biophysical realism. Even as neuron models are simplified and approximated, the neural regions of interest may require an unreasonable amount of running time. To further drive computational neuroscience research, computer scientists and engineers have created more optimized simulation programs and more advanced hardware architecture, respectively.

Biologically, most simulation environments already have built-in spiking neuron models. These models, described as hybrid systems, satisfy a set of differential equations that describe the continuous evolution of several state variables and discrete events (Brette and Goodman, 2012b). The well-known ones are Hodgkin-Huxley (HH), Izhikevich (IZH), and leaky integrate-and-fire (LIF) neuron models. The HH model quantifies the process of spike generation with a set of four differential Equations (Trappenberg, 2010), formalizing their measured findings of the giant axon of a squid. This model uses the voltage dependence and the dynamics of Sodium and Potassium channels, which captures many biological details while losing computational efficiency. The IZH model is a powerful engine, capable of replicating much of the dynamics phenomena observed in neurons. It uses a mathematical formulation derived from the treatment of a neuron as a dynamical system, resulting in a membrane voltage expression (Izhikevich, 2003). This is an intermediate model, which is computationally efficient while still capturing a large variety of response properties of real neurons. The LIF model is comprised of a subthreshold leaky-integrate dynamic, a firing threshold, and a reset mechanism, which gives an approximation of the subthreshold dynamics of the membrane potential with a simple linear differential Equation (Trappenberg, 2010). It is beneficial for analytic calculations and is efficient in numerical implementations. However, the approximation is not sufficient to include most of the response patterns seen in real neurons.

Computationally, most simulators [e.g., NEURON (Carneval and Hines, 2012), NEST (Diesmann and Eppler, 2012), GENESIS (Bower and Beeman, 2012a,b), BRIAN (Brette and Goodman, 2012a)] were designed to run one or more of these models on a single Central processing Unit (CPU). Over the years, they have evolved to support simulations on multiple CPUs for extensibility and higher performance. These enhancements, in combination with parallel computing (Bower and Beeman, 1998; Migliore et al., 2006), have become a necessity to cope with the higher computational and the communication demands of neuroapplications. Recently, a number of developers have investigated the possibility of simulating spiking neural networks on a single Graphical Processing Unit (GPU) (Bernhard and Keriven, 2005; Fernandez et al., 2008; Fidjeland et al., 2009; Nageswaran et al., 2009a,b; Tiesel and Maida, 2009; Bhuiyan et al., 2010; Fidjeland and Shanahan, 2010; Han and Taha, 2010a,b; Hoffmann et al., 2010; Mutch et al., 2010; Scorciono, 2010; Yudanov et al., 2010; Nowotny, 2011; Ahmadi and Soleimani, 2011; Igarashi et al., 2011; Thibeault et al., 2011; Wang et al., 2011) or on multiple Graphics Processing Units (GPUs) (Brette and Goodman, 2012b). All these current simulators have shown significant improvements over their CPU only counterparts by integrating the utilization of GPUs. However, these approaches have had limitations. Two of the main limitations are that researchers either utilize an Izhikevich neuron model while running the simulation on GPU, or if they utilize more than one neuron model (e.g., HH and IZH) their model focuses on small-scale networks. Additionally, they are not capable of running simulations on heterogeneous cluster of GPUs.

To reduce execution times without sacrificing biological details, we have developed a new version of our brain simulator. Here, we present a new CPU/GPU Simulation Environment for Large-Scale Neural Modeling, called the NeoCortical Simulator (NCS) version 6. Previous versions of NCS were designed to run on a CPU or cluster of CPUs. Every version of NCS has implemented a hybrid spiking neuron model. Sub-threshold dynamics are determined by channels that follow the HH formalism. When the voltage crosses a specified threshold value, the membrane potential follows a user-specified spike shape pattern, similar to an LIF neuron. This hybrid model is referred to as an LIF model in the rest of this paper. For a detailed history of NCS and related equations, please refer to our Brain Computation Laboratory's website: http://www.cse.unr.edu/brain/. In addition to the hybrid LIF spiking neurons, NCS6 implements the simplified IZH Equations (Izhikevich, 2003) as a separate neuron type. The Compute Unified Device Architecture (CUDA) by NVIDIA (NVIDIA, 2013) provides an instruction set and tools to developers to work in a GPU environment. NCS utilizes CPUs and CUDA-capable GPUs for simulation. Computationally, shared-memory multiprocessor architectures and recent experiments with clustered GPUs indicate that we will soon be able to simulate a million cells in real time without sacrificing biological detail. In this manuscript, Section 2 explains how NCS has been designed, Section 3.1 gives a validation of its implementation, and Section 3.2 shows a representation of its high performance. Furthermore, we provide a brief comparison between NCS and other simulation environments in Section 3.4. In Section 4 we conclude with a summary of the paper and our planned future work.

## Design

### Simulation composition

At the detailed level, every simulation is comprised of four primary types of elements: neurons, synapses, stimuli, and reports. Neurons represent the cell body and must report two values at each time-step: the spiking state and the membrane voltage. Synapses represent a unidirectional connection between a presynaptic neuron and a postsynaptic neuron. When the presynaptic neuron fires, the synapse introduces a synaptic current into the postsynaptic cell after some specified delay. Stimuli are connected to a neuron and represent a type of external input, able to either clamp the membrane voltage to some level or inject some amount of current. Reports connect to a set of elements (e.g., cell group) and are used to extract output information (e.g., voltage) from those elements and generate the result to some arbitrary data sink.

While each component type has some required constraints, the majority of the internal behavior is determined by the more specific subtype being simulated. For example, one neuron could be specified to simulate a LIF neuron while another neuron could be specified to simulate an IZH cell. The underlying equations governing the behavior are completely different between the two, but they can still be used within the same simulation. The only requirements are that they each receive an external stimulation and/or a synaptic current, and that they each report a firing state and/or a voltage.

### Simulation environment and distribution

To improve the simulation run times, NCS6 is designed to run on clusters of multiple machines, potentially with different computing devices in each computer. These devices include CUDA-capable GPUs, and CPUs. Even within the same device class, the computational power of different devices can be drastically different. To better facilitate load-balancing, a relative computational power rating is assigned to each device. The current method for determining this quantity is to multiply the device's clock rate by the number of computational cores.

**Figure 1:**
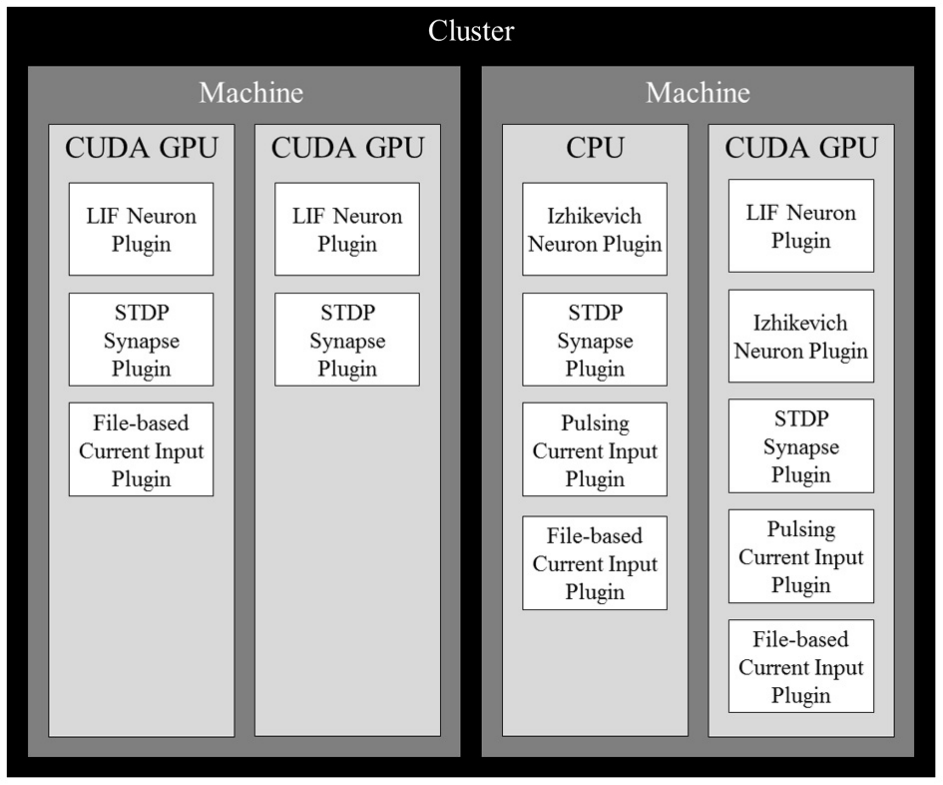
**An example of a complete distribution of simulation elements in NCS6.** Elements are distributed across devices based on the devices' computational power and their dependencies. Synapses and inputs associated with a particular neuron are linked to it on a device. Within devices, elements are organized into contiguous sections by type that are simulated by plugins of their specific type.

After determining the computational power of each device, a cost estimate for each neuron is computed. Since the number of synapses outnumbers the number of neurons and inputs by several orders of magnitude, we use the number of synapses as the cost estimation. Neurons are then sorted in decreasing order of computational cost and distributed across all available computing devices in the cluster so the device with the lowest computational load (total computational cost/computational power) receives the next neuron. Once all neurons in the simulation are distributed, all contributing synapses and stimuli are also placed on the same devices as their targeted neurons. With all elements distributed across all devices, they are further partitioned by their subtypes, each of which being managed by a plugin. Figure 1 shows an example of a complete distribution.

### Data scopes and structures

Due to the distributed nature of NCS6, elements may be referenced in a number of scopes that mirror the environment's hierarchy: plugin, device, machine, and global (cluster). After the distribution is finished, every element is assigned a zero-based ID for each scope. IDs are padded between plugins so that data words for structures allocated in other scopes are related to only one plugin. In general, this means that IDs are padded to a factor of 32 (the number of bits in a word) between plugins. It is important to note that IDs are only unique within the same element type; that is, there can be both a neuron and a synapse with a global ID of 0.

Depending on which elements need access to other elements, certain key data structures are allocated and accessed using different scopes. Data that is specific to an element subtype is stored at the plugin scope. Because synapses may need to access the membrane voltage from their postsynaptic neurons in order to determine their synaptic current contributions, membrane voltages are stored and accessed using device level IDs. The reason is all postsynaptic neurons and their associated synapses reside on the same device due to the way they are distributed. However, because the spiking state of a synapse depends on the spiking state of the presynaptic neuron, the spiking state of neurons is accessed using a global level ID when updating synaptic spiking states.

### Simulation flow and parallelization

The basic flow of a simulation is as follows: for each time-step, the current from stimuli and synapses is computed and used to update the state of every neuron. The resulting spiking state of each neuron is then used to determine the spiking state of their associated synapses in later time-steps.

**Figure 2:**
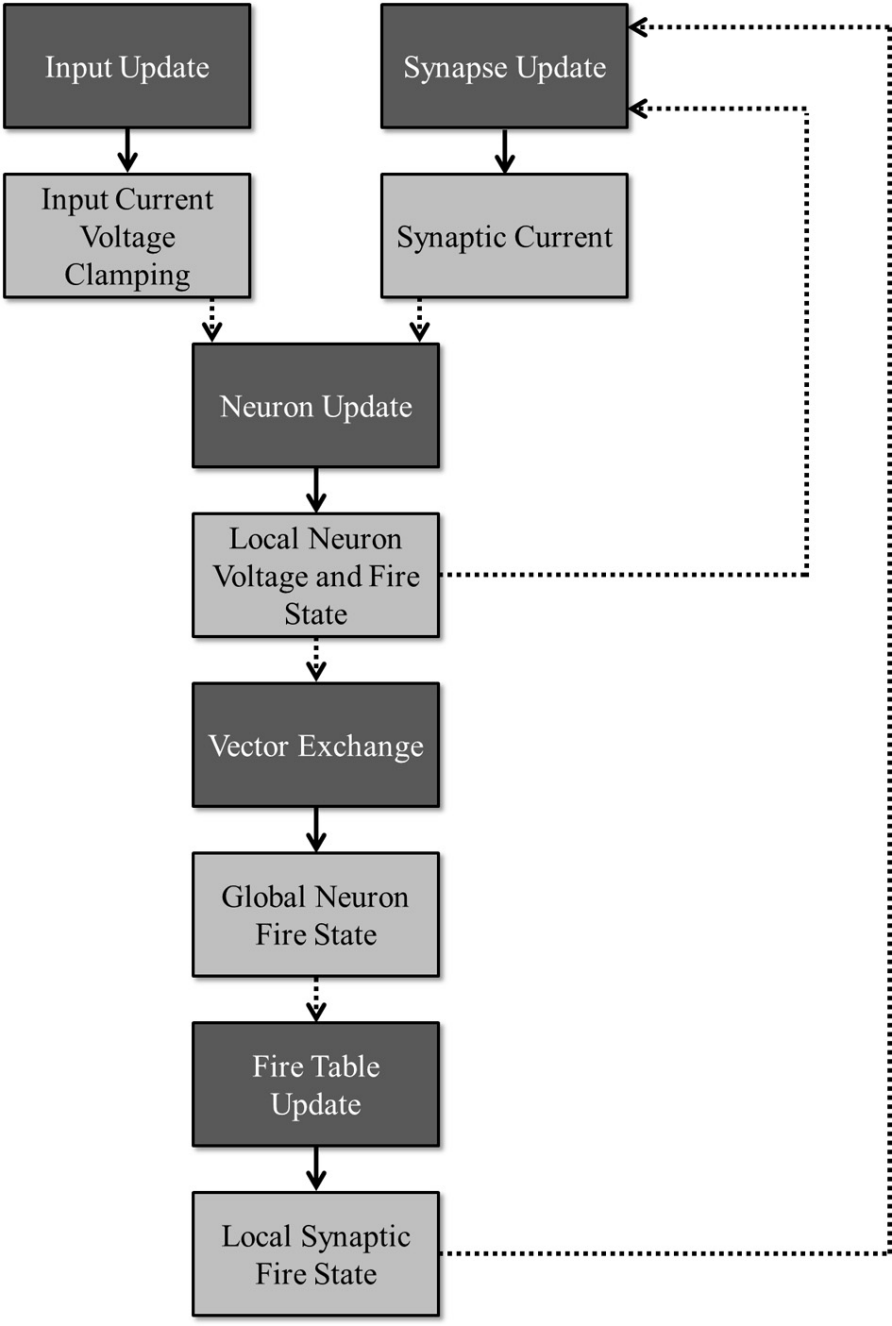
**Division of work: the dark boxes represent stages that can run concurrently as long as the necessary data has been received for a given time step.** Each stage produces an output (denoted by the lighter boxes) that is consumed by the stage denoted by the dotted arrows.

To facilitate maximum utilization of computing devices, the simulation is partitioned into several stages that can be executed in parallel as long as the requisite data for a given stage is ready. Figure 2 illustrates this division of work (dark boxes) along with the required data (light boxes) needed to simulate a particular stage and the data that is produced once that stage has been updated. A publisher-subscriber system is used to pass data buffers from one stage to the next. During the simulation, a stage attempts to pull all necessary data buffers from their associated publishing stages. The stage is blocked until all the data is ready. Once it obtains all the required data buffers, it advances the simulation by a single time-step and publishes its own data buffer while releasing all the others that it no longer needs. When all subscribers to a data buffer release it, the data buffer is added back to its publisher's resource pool for reuse. For any given stage, a limited number of publishable buffers are used to prevent a stage from consuming all computational resources and getting unnecessarily ahead of any other stages. For example, without limiting the buffer count, because the input update stage requires no data from any other sources, the stage could simulate all time-steps before a single neuron update is allowed to occur, effectively adding a serial time cost to the overall run time.

Within a single stage, further granularity is gained by parallelizing across subtypes. As an example, if a device simulates both LIF Neurons and Izhikevich Neurons, the plugins updating each can be executed in parallel. Due to padding from the ID assignments, updates should affect completely separate regions of memory, including operations on bit vectors. Exceptions to this, such as when an input writes to a device-indexed input current for its target neuron, are handled by using atomic operations or by aggregating partial buffers generated by each plugin. The method chosen depends on the type of device and its memory characteristics. While plugins are allowed to update ahead of one another, the results for from a stage at a given time-step will not be published to subscribers until all plugins (in that stage) have updated up to that time-step.

#### Input update

The purpose of the input update stage is to compute the total input current to each neuron on the device as well as any voltage clamping that should be done. The input current is represented by an array of floating point values, one for each neuron (including padding) on the device, initialized to zero at the beginning of each time-step. The voltage neurons are clamped and stored in a similar fashion where a bit vector is used to select which neurons should actually be clamped.

Inputs are expected to be updated by input plugins designed to handle their subtype. Other than the device-level Neuron ID for each Input that is statically determined at the beginning of the simulation, input plugins rely on no other data from any other stage of the simulation. As such, they are allowed to simulate ahead of the rest of the system as long as it has empty buffers that can be written to and published.

#### Neuron update

Unlike the input update stage, the neuron update stage has two dependencies: the input current per neuron published from the input update stage and the synaptic current per neuron published by the synapse update stage. Given these two pieces of information, this stage is expected to produce the membrane voltage and spiking state of every neuron on the device. Like the input current, the membrane voltage is represented by an array of floating point values. On the other hand, the spiking state is represented by a bit vector.

Similar to inputs, neurons are expected to be updated by neuron plugins designed to handle their subtypes. Despite receiving and writing data out into device-level structures, neuron plugins operate purely in plugin space. This is possible due to the fact that each plugin is given a contiguous set of device-level IDs during the distribution. As a result, device-level data passed into each plugin is simply offset accordingly to yield the appropriate plugin-level representation.

#### Vector exchange

The result of the neuron update stage is the firing state of every neuron residing on the device. However, synapses are distributed purely based on the postsynaptic neurons and as such the presynaptic neurons could possibly reside on a different device. Thus, to determine synaptic spiking, the state of every neuron in the simulation must be gathered first. Again, the publisher-subscriber scheme is used to pass data asynchronously. However, rather than passing data between stages, it is used to pass data between different data scopes.

Figure 2 shows the flow of the neuron spiking information across a cluster. When the device-level vector exchanger receives a local firing vector, the data is published to the machine-level vector exchanger. Within this exchanger, the local vector is copied into a global vector allocated in the system memory. Once all local device vectors are copied for a single time-step, the complete machine-level vector is broadcast to all the other machines in the cluster. After all machines in the cluster finish broadcasting, the complete global firing vector is published back to the device-level vector exchangers where it is copied back into the appropriate type of device memory before being published out to any subscribing stages.

#### Firing table update

With the firing state of every neuron in the simulation, a device can determine when all of its synapses will receive the firing based on a per-synapse delay value. Given the potential range of delays, this information is stored within a synaptic firing table. A row of the table is a bit vector representing the firing state of every synapse on the device. The number of rows in the table depends on the maximum delay of all local synapses. When this stage receives the global neuron fire vector, each synapse checks its associated presynaptic neuron for a firing state. If it is firing, the synapse adds its delay to the current time-step to determine the appropriate vector which is then modified by setting its bit to 1.

After updating the table for a single time-step, the table row associated to that step can be published. However, up to N time-steps ahead of the current time can be published, where N is the minimum delay across all local synapses. This allows devices to simulate ahead of one another to a point rather than being completely locked in step. Additionally, the publication of these extra buffers at the beginning of the simulation allows the data to start flowing through the simulation.

#### Synapse update

Given the firing state of each synapse on the device, the synapses themselves can be updated. Like the input update stage, the synapse update stage produces the total synaptic current per device-level neuron also represented by an array of floating point values. In terms of operating spaces, synapse plugins update synapses that operate at both the plugin and device levels, reading from the synaptic fire vector while writing to the synaptic current vector.

#### Reports

Reports gather information regarding some aspect of the simulation. They are specified by the user as a set of elements and values that should be extracted from them as the simulation progresses. Because these elements can be scattered across multiple devices and/or different machines and the data required can reside on one of several different scopes, every machine, device, and plugin are given a unique identifier. Following the distribution, every element that must be reported on can be located by the appropriate ID based on the data scope and the identifier within the data source.

With these two values, the appropriate data can be extracted during the simulation. To accomplish this, a single reporter is instantiated on each machine, which contains at least one element that should be collected. Then, a reporter subscribes to each publisher of the data through a more generalized publisher-subscriber interface. This interface allows a reporter to access data arrays along with the memory type using a string identifier. At each time-step, the reporter extracts data from all of its subscriptions and aggregates them as necessary. A separate MPI communication group is then used to further aggregate these data across the entire cluster asynchronously before being written out to a file or some other data sink.

Instead of using a built-in reporter type, a plugin-type interface is devised to provide flexibility in terms of data extraction, aggregation, communication, and output techniques without overly complicating the resulting code. For instance, a reporter that counts the number of neuron firings may choose to minimize data bus traffic on CUDA devices by implementing the count directly on the device and retrieving the single value rather than by downloading the entire buffer to the system memory first before operating on it. Implementations of the reporter interface are given access to an MPI communication group along with the element IDs and source identifiers to accomplish the aforementioned tasks.

### CUDA implementation

Every CUDA plugin in any stage of the simulation flow uses a separate CUDA stream to enqueue work for the GPU, sleeps while waiting for kernel execution to finish, and publishes the results to subscribing stages when the results are ready. Each stream operates independently on separate pieces of data, allowing the CUDA scheduler to execute kernels from different streams concurrently in order to maximize hardware utilization.

Unlike the computationally-straightforward Izhikevich model, the LIF model as specified by NCS presents a number of challenges when implementing it in CUDA. To begin with, LIF neurons can be composed of multiple compartments that affect one another and have different synaptic connections. To maintain minimal data transfer, all compartments of a single LIF neuron are decomposed into neuron-like objects that must be distributed to the same device, localizing cross-compartment interactions to that device. Since each compartment is modeled like a neuron, compartment-specific connections are realized as well.

An additional complexity of the LIF neuron comes from the ability for a compartment to have one or more channels that alter its current based on a number of different attributes. The solution to this comes from applying the simulation flow breakdown to this smaller subproblem. Each unique channel type is implemented as a plugin to the larger LIF plugin in order to minimize branching within a single kernel. At each time-step, the channel plugins concurrently modify a current buffer. This buffer is then published to the compartment updater, which in turn publishes the compartments newly updated state for use by the channel plugins in the subsequent time-step.

A final challenge to modeling NCS neurons is due to the behavior of firings. Rather than sending a single impulse across a synapse when the neuron fires, a waveform is sent over a potentially large number of time-steps. Repeated firings over a short time period produce multiple waveforms that are summed together. To enable this memory of firings in CUDA, the synaptic update plugin behavior is decomposed into a few steps. A synapse begins by checking the fire table to see if a firing has been received. If so, it pushes the event composed of a waveform iterator onto a list. That list along with the list from the previous update are then updated, computing the total synaptic current for a single neuron at the same time. If an event has not yet iterated across its entire waveform, it is pushed onto a new list that is published for the next time-step.

## Results

The results of this manuscript are presented in the form of: neuron model validation, NCS performance, existing models using NCS, and a comparison of simulation environments.

### Neuron model validation

**Figure 3:**
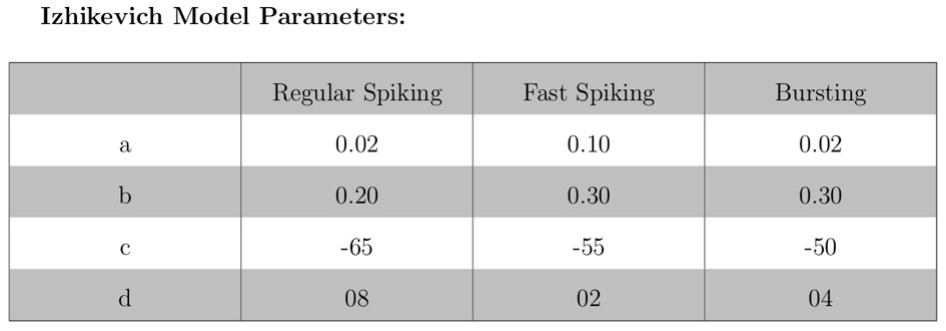
**IZH neuron model: specific values used for parameters a, b, c, and d**.

The validation of our neuron models is crucial to the reliability of modeling studies. We have compared membrane potential traces using our two types of neurons models in response to current injection with electrophysiological data (Contreras, 2004) and the well-known Izhikevich firing patterns (Izhikevich, 2003). As examples, we looked at three major types of neuronal firing patterns: regular spiking (RS), fast spiking (FS), and bursting (B). For the LIF neuron model, we used different types of channels and parameters. Channels included voltage-dependent and calcium-activated potassium channels. For the IZH neuron model, we used specific values for the parameters a, b, c, and d, which are given in Figure 3.

**Figure 4:**
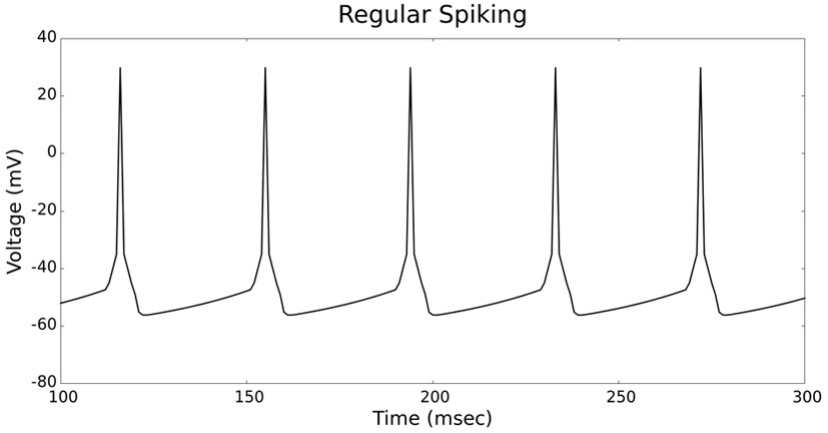
**LIF neuron model: regular spiking firing patterns**.

**Figure 5:**
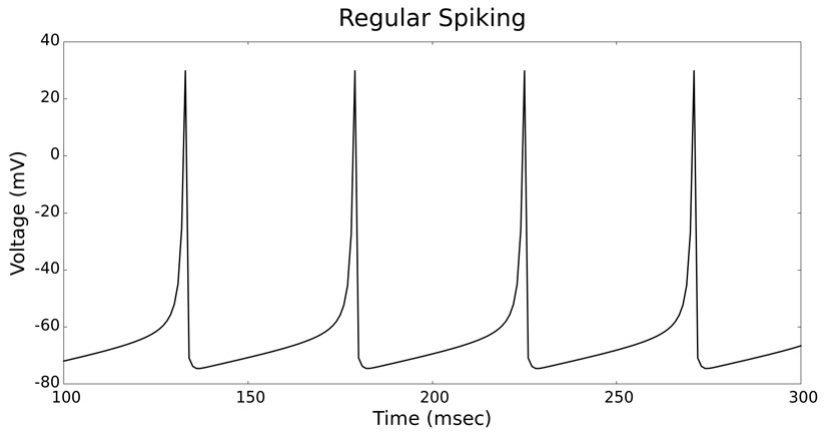
**IZH neuron model: regular spiking firing patterns**.

**Figure 6:**
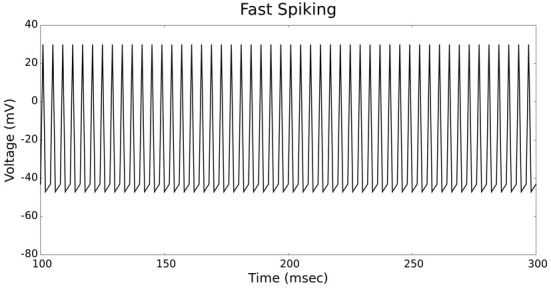
**LIF neuron model: fast spiking firing patterns**.

**Figure 7:**
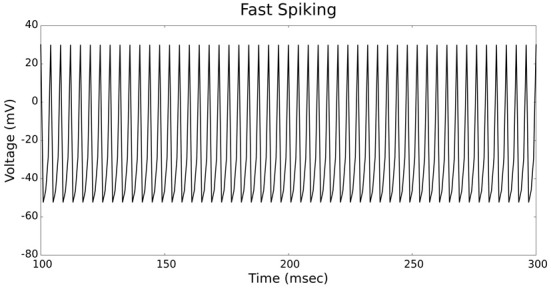
**IZH neuron model: fast spiking firing patterns**.

**Figure 8:**
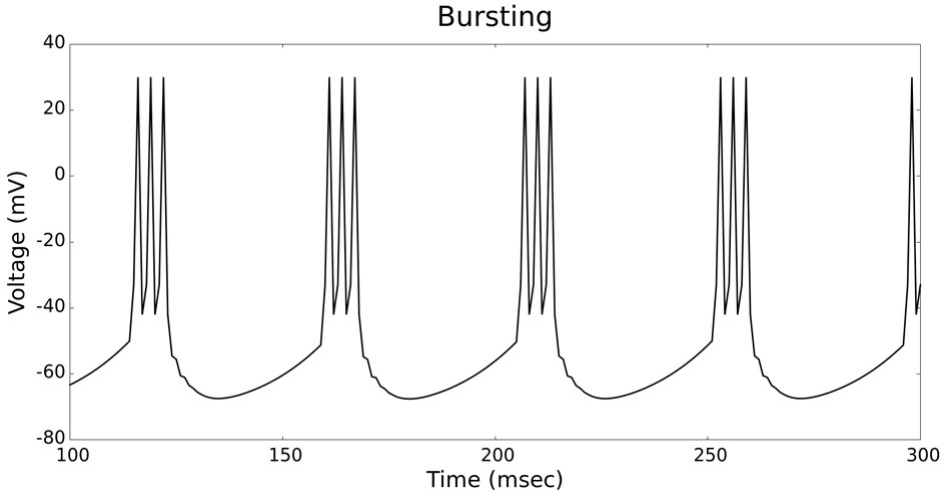
**LIF neuron model: bursting firing patterns**.

**Figure 9:**
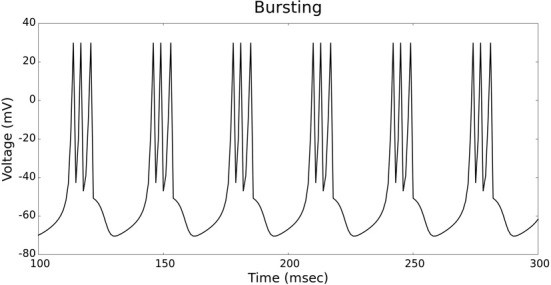
**IZH neuron model: bursting firing patterns**.

Figures 4, 5 show the firing patterns of simulated regular spiking neurons using the LIF and the IZH neuron models, respectively. Figures 6, 7 show the firing patterns of simulated fast spiking neurons using the LIF and the IZH neuron models, respectively. Figures 8, 9 show the firing patterns of simulated bursting neurons using the LIF and the IZH neuron models, respectively. All six figures graph a sample of the simulation from 100 to 300 msec. Overall, our two neuron models were validated by closely replicating spike shapes and spike frequencies from electrophysiological data (Contreras, 2004) and the well−known Izhikevich firing patterns (Izhikevich, 2003) for three major types of neurons: RS, FS, and B. Note: our two models are not limited to these three types; all neural patterns can be replicated.

### NCS performance

**Figure 10:**
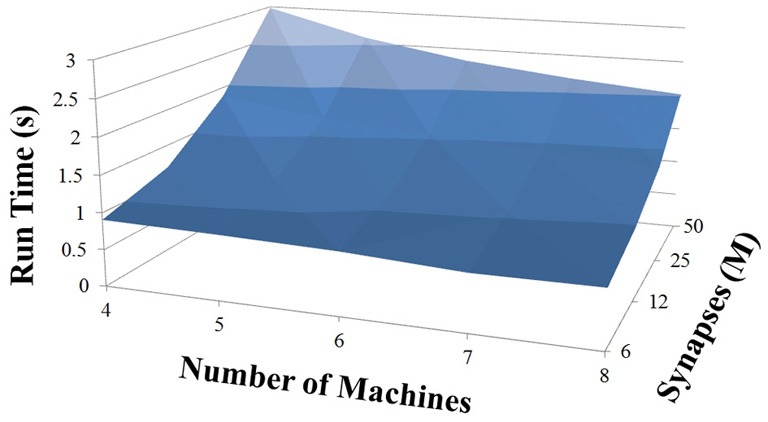
**LIF neuron model: 1 s simulation**.

**Figure 11:**
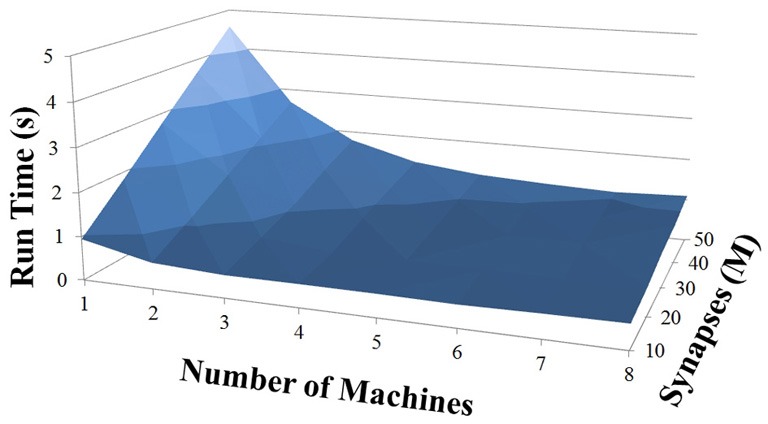
**IZH neuron model: 1 s simulation**.

**Figure 12:**
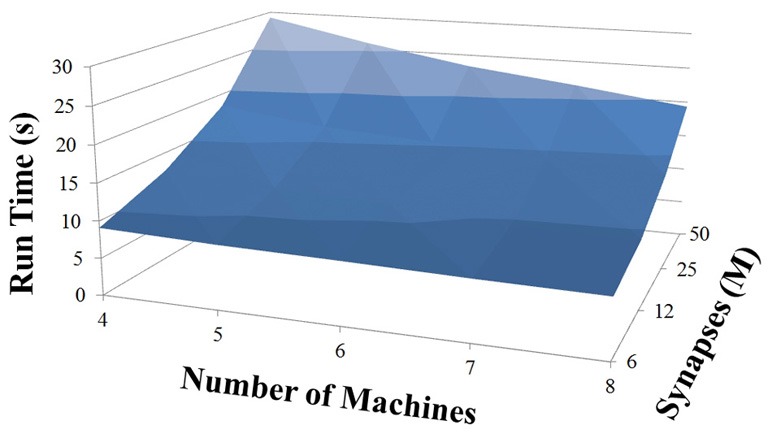
**LIF neuron model: 10 s simulation**.

**Figure 13:**
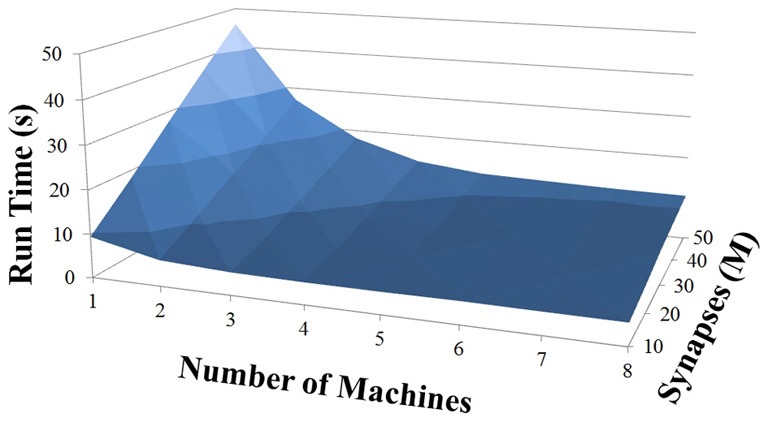
**IZH neuron model: 10 s simulation**.

Based on recent development and enhancements of NCS, we are capable of running large-scale neural models (100,000–1,000,000 neurons) faster than most simulators by distributing data across multiple GPUs. Considering a synapse to neuron ratio of 100 (e.g., 500,000 neurons and 50 million synapses), NCS runs any models up to almost 1 million neurons in real-time, for example, 1 s simulation = 1 s (IZH) or 2 s (LIF) real-time, as presented in Figures 10, 11 for the hybrid and Izhikevich neurons, respectively. In the NCS performance figures, eight machines were used with each having two video cards (GTX 680s, GTX 480s, GTX 460s, or Tesla C2050s) with a time-step of 1 ms. From one to ten-second simulations, NCS has shown no loss of performance over time, as shown in Figures 12, 13. However, the loss of performance can occur in models containing more than 50 million synapses due to the high computation power required by synapses. The limit in terms of communications occurs when the size of the neuron vector is too large for the network to handle. In the case of GigE(1000 Mbps) simulating at 1 ms intervals, we have 1 Mb per update, which represents 1 million cells (1 bit per cell). Additionally, there is MPI packet overhead. Currently, the main reason for loss of performance in very large models is due to memory constraints of the GPUs and not due to network limitations.

### Existing models using NCS

For details regarding existing models using NCS, related research projects, and publications please refer to our Brain Computation Laboratory's website: http://www.cse.unr.edu/brain/.

### Comparison of simulation environments

**Figure 14:**
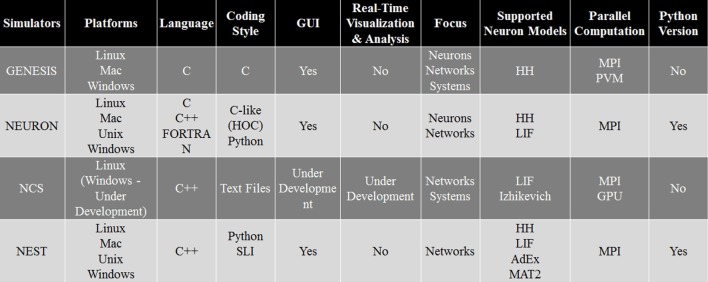
**Simulation environments comparison**.

As every simulation environment have their own advantages and disadvantages, we have compared NCS with three well-known simulators, NEURON, GENESIS, and NEST. This comparison, presented in Figure 14, can be useful for scientists to decide which simulator is better suited for their modeling experiments. Specifically, it describes the four simulation environments' features, such as platforms, back-end language, front-end coding style, GUI, appropriate applications, supported neuron models, type of parallel computation, and possible python version. Overall, NCS is currently well suited for large-scale neural networks and average biological details which can be simulated with LIF and IZH models. The input language for NCS is a text file and it requires minimum computer programming experience.

## Discussion and future work

NCS6 is a new, free, open-source, parallelizable, and scalable simulator, designed to run on clusters of multiple machines, potentially with high performance computing devices in each of them. Simulator, tutorial slides, models, documentation, and conference posters are available for download at http://www.cse.unr.edu/brain/ncs. It has built-in LIF and IZH neuron models that replicate biological neural firing patterns based on experimental data (Contreras, 2004). All firing patterns can be reproduced with realistic spikes shapes and spikes frequencies. If users are not satisfied with these available models, they also have the flexibility to design their own plug-in interfaces for different neuron types. NCS6 is currently able to simulate one million cells and 100 million synapses in quasi real time by distributing data across these heterogeneous clusters of CPUs and GPUs. A variety of models have been created and simulated with NCS, and they have shown interesting findings on high-level behaviors (e.g., navigation). The advantages of using NCS6 are its computational power, its biological capabilities at multiple levels of abstraction, and its minimum computer programming demand. NCS6's main limitations include its lack of biophysical parameters, its only availability on LINUX platforms, and the absence of a GUI. Therefore, our current work consists of increasing the biological details behind NCS6 without affecting simulation time. NCS6 should be soon available on Windows, and be able to run on openCL-capable devices. Additionally, our main focus has been on developing a real-time visualization and analysis tool to make the use of NCS6 convenient to a broader community.

### Conflict of interest statement

The authors declare that the research was conducted in the absence of any commercial or financial relationships that could be construed as a potential conflict of interest.

## Appendix

### NCS cell equations

At a cellular level, NCS solves a limited and slightly reordered form of the Hodgkin-Huxley model that is similar to Equation (1). However, during the numerical integration a constant membrane leak is added. This is explained further below.

(1) $$C_{N}\frac{dV}{dt}-I_{M}-I_{A}-I_{AHP}-I_{input}-I_{syn}+I_{leak}=0$$

The currents expressed in this equation fall into several different categories that are only briefly explained here. To begin, both *I*_*M*_ and *I*_*AHP*_ contribute to the membrane voltage by controlling spike-frequency adaptation. These are small ionic currents that have a long period of activity when the membrane voltage is between rest and threshold. *I*_*M*_ is the Non-inactivating Muscarinic Potassium Current and is defined by

(2) $$I_{M}={\bar{g}}_{M}Sm^{P}(E_{k}-V)$$

Where S is a non-dimensional strength variable added to NCS and *P* is the power that the activation variable *m* is raised to. This is essentially decreasing the slope of the activation variable. The change of that activation variable is defined as

(3) $$\frac{dm}{dt}=\frac{m_{\infty }-m}{\tau_{m}}$$

where

$$\begin{array}{l} \tau_{m}=\frac{\epsilon }{e^{\left(\frac{V-V_{1/2}}{\omega }\right)}+e^{-\left(\frac{V-V_{1/2}}{\eta }\right)}}\\ m_{\infty }=\frac{1}{1+e^{-\left(\frac{V-V_{1/2}}{\xi }\right)}} \end{array}$$

ϵ is the scale factor.

*V*_1/2_ satisfies the equation *m*∞(*V*_1/2_) = 0.5.

ω, η, and ξ are slope factors affecting the rate of change of the activation variable m.

Notice that (2) is different from the traditional equation shown below in equation (4). This reverse of the driving force explains the sign changes in equation (1).

(4) $$I_{M}={\bar{g}}_{M}m_{m}\left(V-E_{K}\right)$$

*I*_*AHP*_ is the current provided by the other small spike-adaptation contributing channel. These are voltage independent potassium channels that are regulated by internal calcium.

(5) $$I_{AHP}={\bar{g}}_{AHP}Sm^{P}(E_{K}-V)$$

Where S is a non-dimensional strength variable added to NCS and *P* is the power that the activation variable *m* is raised to. The change of that activation variable is defined as

(6) $$\frac{dm}{dt}=\frac{m_{\infty }-m}{\tau_{m}}$$

$$\tau_{m}=\frac{\epsilon }{f(Ca)+b}$$

$$m_{\infty }=\frac{f(Ca)}{f(Ca)+b}$$

Where

ϵ is a scale factor.

*b* is the backwards rate constant, defined as CA_Half_Min in the NCS documentation.

*f*(*Ca*) is the forward rate constant defined by (7).

(7) $$f(Ca)=\kappa {\left[Ca\right]}_{i}^{\alpha }$$

Internal calcium concentrations are calculated at the compartment level in NCS. Physiologically the calcium concentration of a cell increases when an action potential fires. After the action potential has ended the internal concentration of calcium will diffuse throughout the cell where it is taken up by numerous physiological buffers. In NCS this diffusion/buffering phenomena is modeled by a simple decay equation defined by Equation (8).

(8) $${\left[Ca\right]}_{i}(t+1)={\left[Ca\right]}_{i}\left(t\right)\left(1-\frac{dt}{\tau_{Ca}}\right)$$

Where

*dt* is the simulation time step.

τ_*Ca*_ is the defined time constant for the Ca decay.

When an action potential fires in NCS the internal calcium concentration is increased by a static value specified in the input file.

The third and final channel type modeled in NCS is the transient outward potassium current or *K*_*a*_. This channel requires hyperpolarization for its activation; meaning that the channel will open during inhibitory synaptic input. This is defined by (9).

(9) $$I_{K}={\bar{g}}_{M}Sm^{P}h^{C}\left(E_{K}-V\right)$$

Where as before S is a non-dimensional strength variable added to NCS, *P* is the power that the activation variable *m* is raised to and *C* is the power that the inactivation variable *h* is raised to. The change of activation and inactivation variables is defined by (10) and (11).

(10) $$\frac{dm}{dt}=\frac{m_{\infty }-m}{\tau_{m}}$$

(11) $$\frac{dh}{dt}=\frac{h_{\infty }-m}{\tau_{h}}$$

Where

$$m_{\infty }=\frac{1}{1+e^{-\left(\frac{V-V_{1/2m}}{\xi }\right)}}$$

*V*_1/2*m*_ satisfies the equation *m*∞(*V*_1/2*m*_) = 0.5.

ξ is slope factor affecting the rate of change of the activation variable m.

$$h_{\infty }=\frac{1}{1+e^{-\left(\frac{V-V_{1/2h}}{\eta }\right)}}$$

*V*_1/2**h**_ satisfies the equation *h*∞(*V*_1/2*h*_) = 0.5.

η is slope factor affecting the rate of change of the inactivation variable h.

τ_*m*_ and τ_*h*_ are voltage dependent. NCS allows this dependence to be defined using an array of values for both voltages and time constants. This is defined by (12).

(12) $$\tau \left(V\right)=\{\begin{array}{ll} \tau \left(1\right) & \text{if V}<\text{V}(\text{1}),\\ \tau \left(2\right) & \text{if V}<\text{V}(\text{2}),\\ \vdots & \\ \tau \left(n\right) & \text{if V}<\text{V}(\text{n})\\ \tau \left(n+1\right) & \text{else} \end{array}$$

The leakage current is voltage-independent and is modeled by (13). Notice that the driving force is expressed using the normal convention. This is the reason the leakage current is subtracted in the membrane voltage equation rather than added, as seen in the traditional membrane voltage equations.

(13) $$I_{leak}=g_{leak}\left(V-E_{leak}\right)$$

The synaptic currents are calculated by

(14) $$I_{syn}={\bar{g}}_{syn}PSG\left(t\right)\left(E_{syn}-V\right)$$

The numerical integration scheme employed by NCS is similar to an Eulerian method. However, as mentioned above a constant leak term is added to the discretized form of (1). To begin the current values defined above are summed

(15) $$I_{Total}=I_{M}+I_{A}+I_{AHP}+I_{input}+I_{syn}-I_{leak}$$

The new voltage is then calculated as a combination of the defined membrane resting potential, the previously calculated membrane potential, the membrane resistance, capacitive time constant and total currents.

(16) $$V\left(t+1\right)=V_{rest}+\left(V\left(t\right)-V_{rest}\right)\left(1-\frac{\Delta }{\tau_{mem}}\right)+\Delta \frac{I_{Total}}{C_{n}}$$

Rearranging for clarity

(17) $$V\left(t+1\right)=V\left(t\right)+\left(V_{rest}-V\left(t\right)\right)\frac{\Delta }{\tau_{mem}}+\Delta \frac{I_{Total}}{C_{n}}$$

Where

$C_{n}=\frac{\tau_{mem}}{R_{mem}}$

*R*_*mem*_ is the defined resistance of the membrane.

τ_*mem*_ is the defined capacitive time constant of the membrane.

Δ is the change in time between *t* and *t* + 1.

Notice the form of (1) in a simple Eulerian integration scheme would be

(18) $$V\left(t+1\right)=V\left(t\right)+\Delta \frac{I_{Total}}{C_{n}}$$

The addition of the middle term in Equation (17) numerically drives the membrane voltage of the cell back to a predefined resting potential.

When the voltage crosses a specified threshold value *v*_*threshold*_, the membrane potential follows a user-specified spike shape pattern. During this time, the internals of each channel are updated; however, they have no effect on the value of the memberane potential. At the end of the pattern, calculations resume using Equation(17).
